# Reflux Revisited: Advancing the Role of Pepsin

**DOI:** 10.1155/2012/646901

**Published:** 2011-11-10

**Authors:** Karna Dev Bardhan, Vicki Strugala, Peter W. Dettmar

**Affiliations:** ^1^Rotherham General Hospital, Moorgate Road, Rotherham, South Yorkshire S60 2UD, UK; ^2^Technostics Ltd., The Deep Business Centre, Kingston Upon Hull, East Yorkshire HU1 4BG, UK

## Abstract

Gastroesophageal reflux disease is mediated principally by acid. Today, we recognise reflux reaches beyond the esophagus, where pepsin, not acid, causes damage. Extraesophageal reflux occurs both as liquid and probably aerosol, the latter with a further reach. Pepsin is stable up to pH 7 and regains activity after reacidification. The enzyme adheres to laryngeal cells, depletes its defences, and causes further damage internally after its endocytosis. Extraesophageal reflux can today be detected by recognising pharyngeal acidification using a miniaturised pH probe and by the identification of pepsin in saliva and in exhaled breath condensate by a rapid, sensitive, and specific immunoassay. Proton pump inhibitors do not help the majority with extraesophageal reflux but specifically formulated alginates, which sieve pepsin, give benefit. These new insights may lead to the development of novel drugs that dramatically reduce pepsinogen secretion, block the effects of adherent pepsin, and give corresponding clinical benefit.

*“For now we see through a glass, darkly.”*

—First epistle, Chapter 13, Corinthians

## 1. Introduction

This quotation from the Bible, often used in drama and thrillers, symbolises “that the clarity of a situation is often obscured”. It is in our view an apt description of the syndrome we today recognise as extraesophageal reflux (EER). The inspired insight in the 1990s that the symptoms and findings might constitute a distinct entity [1] was followed by growing awareness in the last decade that the underlying cause was gastroesophageal reflux. Hence, the Montreal classification included several extraesophageal features within the spectrum of gastroesophageal reflux disease (GERD), the association considered “established” for laryngeal symptoms, cough, and asthma (and “proposed” for recurrent otitis media, idiopathic pulmonary fibrosis, pharyngitis, and sinusitis) [2]. This was a remarkable foresight, for at the time, the data on which we today regard for EER was still emerging. Today, however, there is strong evidence that laryngeal damage from EER is mediated by pepsin. This decade may see these discoveries lead to clearer understanding of the disease process and consequently lead to the development of effective therapy.

The earlier confusion surrounding the entity of EER and the story now unfolding is reminiscent of the early 1980s when an unusual organism came to be identified in the stomach, particularly in those with peptic ulcer. Looking back, it may seem self-evident that *Helicobacter pylori* (*H. pylori*) was closely related to the development of duodenal ulcer (DU)—but it certainly did not seem so at the time.

### 1.1. Acid—and Beyond

GERD has, with good reason, been widely regarded as the consequence of excessive reflux from the stomach into the lower esophagus, the *acid* component of the refluxate damaging the esophageal mucosa. This link is made indelible in the clinician's mind by the rapid and sometimes dramatic relief proton pump inhibitors (PPIs) give, through profound acid suppression. Today, we are increasingly aware reflux can reach much further, extending beyond the upper esophagus into the pharynx, larynx, airways, and middle ear, and may damage these structures [3, 4].

The benefits of the PPIs are striking in patients with *typical* GERD, that is, those with lower esophageal symptoms, principally retrosternal burning (“heartburn”), and regurgitation (the two together commonly referred to as the “classical symptoms” of reflux) with or without erosive esophageal changes. This is reflected by numerous clinical trials and reinforced by worldwide experience [5, 6]. In contrast, PPIs generally give little benefit when symptoms arise from refluxate-damaged organs further away, indicating that unlike in the esophagus, acid may not be the damaging agent. In EER, the damaging agent we suggest is pepsin (and perhaps bile acids).

Pepsin is produced only in the stomach; hence evidence of its presence in these organs signifies refluxate has reached them. The enzyme has recently been identified within the laryngeal epithelium, in the saliva of patients with suspected reflux laryngitis, and in the exhaled breath of those with airways and lung disease, where reflux is thought to have played a part. Pepsin has also been found in the middle ear in otitis media effusions (where bile acids, too, have recently been identified).

“Host factors” too are presumably involved which influence who develops disease and how severe it becomes. These factors may also have a bearing whether the disorder manifests with typical reflux symptoms arising from the esophagus or as EER. Many with EER have little or no retrosternal burning or regurgitation; this is surprising, for to reach the extraesophageal areas, the refluxate would first have to travel through the esophagus. Refluxate is liquid, but there is growing awareness that it may also be an aerosol. Firm evidence is, however, yet to emerge. Nevertheless, an aerosol remains an “attractive” possibility for it would account for observations as yet unexplained. For example, intuitively, it would seem that the concentration of hydrogen ions would be less in an aerosol (compared to that in liquid refluxate), perhaps below a threshold to trigger esophageal symptoms.

The concept of pepsin and bile acids playing a role in reflux disease developed many years ago but has been often overlooked in recent times, as the very success of PPIs increasingly focused attention on acid (to the exclusion of other factors) but also in part because the relevant literature is not usually referred to by gastroenterologists.

## 2. Aim

The *aim* of this paper is, therefore, to draw the key evidence together and to raise awareness of EER amongst gastroenterologists, who today are increasingly invited by ENT and respiratory specialists to help investigate patients in whom this condition is suspected. Bile acids are mentioned but the focus is on pepsin; GERD is often referred to but mainly to compare and contrast with EER, the main thrust.

We have broadly adhered to the philosophy of the Montreal classification of GERD, [2] departing only when it does not sufficiently accommodate more recent findings and evolving concepts of pathophysiology. Thus, a degree of reflux into the lower esophagus and without any symptoms is regarded as physiological gastroesophageal reflux (GER). Gastroesophageal reflux disease (GERD) is the term applied when reflux is accompanied by typical symptoms, with or without erosive mucosal damage. At this stage, the total duration of esophageal acid exposure is considerably longer than in physiological reflux. Whilst extraesophageal reflux disease is increasingly recognised, relatively little is known if “physiological” extraesophageal reflux (EER) occurs.

## 3. History 

### 3.1. GERD: A New Concept Emerges

In 1934, Asher Winkelstein first raised the possibility that the symptoms in five of his patients might have arisen from peptic esophagitis, a condition resulting “from the irritant action on the mucosa of hydrochloric acid and pepsin” [7–9]. From the late 1950s, elegant experimental studies have demonstrated the complex interrelationship between bile acids, pepsin and hydrochloric acid (HCl) interacting and leading to esophageal damage.

### 3.2. Shifting Perceptions

The role of these nonacid factors, however, appeared to diminish in the clinician's perception when the histamine H_2_ receptor antagonists (H_2_RA) emerged in 1976. These were the first drugs to powerfully reduce acid secretion and proved highly effective in controlling peptic ulcer, thus demonstrating the central role of acid in the disease process. By extension, it seemed likely to also be of use in GERD, but the clinical benefits proved to be only modest. Acid inhibition, powerful with the H_2_RA, was profound with the new class of drugs, the proton pump inhibitors (PPIs), which became available in 1989. These drugs proved markedly superior to H_2_RA in the treatment of GERD, which reinforced the growing perception that it was the *acid* component of refluxed gastric contents that was the cause of esophageal damage.

### 3.3. Pepsin and Bile Acids: At the Beginning

#### 3.3.1. Pepsin

The elegant experimental studies of Goldberg et al. [10] clearly demonstrated pepsin can damage the esophagus. Cat esophagi were infused for an hour with HCl, the pH ranging from 1 to 2.3. Acid at pH 1 proved very damaging, whereas at pH 2.3 was without effect; adding pepsin to each of these infusates caused no further damage. In the intermediate acidity range of pH 1.6 and 2.0, however, the damage was proportional to the amount of pepsin added (25 and 50 *μ*g/mL). Blocking the enzyme effect by first premixing with amylopectin sulphate (a synthetic pepsin inhibitor) protected the esophagus, thereby confirming that pepsin can, in the appropriate circumstances, cause damage.

HCl at pH 1 is probably not encountered in the gastric lumen (other than in exceptional circumstances), for the secreted acid is rapidly diluted. A pH of 1.6–3 is common, however, and it is in this range when gastric refluxate containing acid and pepsin is most damaging to the esophageal mucosa. Pepsinogen arises from the gastric peptic cells (also called the chief cells) which share space in the same glands as the acid-secreting parietal cells: the two secretions are independently controlled but almost always occur together. Reflux, therefore, irrespective of its pH, always contains pepsin (see below).

#### 3.3.2. Bile Acids

Experimental studies in the 1980s demonstrated the role of bile acids in damaging the esophageal mucosa. In a series of studies by Harmon and colleagues [11] and by Schweitzer et al. [12], varying concentrations of taurine-conjugated and unconjugated bile salts were infused into rabbit esophagi at pH 2, 5 and 7. Significant disruption of the esophageal mucosal barrier occurred at bile acid concentrations similar to those in the stomach of patients with esophagitis [13]. The evidence, however, suggested that such disruption was probably not the direct effect of bile acids solubilising the cell membrane phospholipids. Instead, bile acids enter the epithelial cells and disrupt cellular machinery from within, hence interfere with the cell barrier function. Such entry is determined by the physicochemical properties of the bile acids. Taurine-conjugates have a pKa of ~2 that is, half the molecules are in solution at pH 2 and, being charged, cannot penetrate the lipid bilayer of the cell membrane. As the pH progressively rises, more bile acids come out of solution and by pH 7 are insoluble, lose their charge and as a result can now enter the epithelial cells.

These experimental studies have particular clinical relevance, for bile is present in esophageal refluxate [14–16] and is most frequently noted in patients with severe esophagitis or complicated Barrett's esophagus [17, 18] in whom it is present at high concentrations [19].

### 3.4. Conceptualizing the Mechanism of Damage

Putting these observations together, one can conceptualise circumstances where pepsin in the refluxate disrupts the esophageal mucosal barrier by acting on the epithelial cell surface, whilst bile acids achieve the same effect by diffusing into the cell and damaging from within.

These seemingly complex mechanisms contrast sharply from the “corrosive” action of acid, an effect “simpler” to picture. Gastric acid consists of H^+^ and Cl^−^ ions in water. When in high concentrations, intuitively, the fluid is more “corrosive”; hence, the longer the time in contact with the esophagus, the greater the likelihood it will damage the mucosa. Conversely, lower concentrations are less damaging. Importantly, the basic constituents of acid are unchanged, only its concentration.

## 4. Pepsin: Nature, Activation, Acidity, and Enzyme Activity: Clinical Significance

### 4.1. Nature and Activation

Pepsin is an ancient molecule and present in all vertebrates studied, such as fishes and mammals. The stomach is largely devoid of live organisms (with the exception of *H. pylori*), a state widely believed to result from the presence of gastric acid, which acts as a “bulk steriliser”. A second important action of acid is the activation of pepsinogen. This releases pepsin which initiates digestion through proteolysis, an action which also probably helps keep the stomach free of most bacteria [20]. 

The peptic chief cells produce and store pepsinogen, the precursor of the active enzyme. Pepsin, an aspartic proteinase, is a large bilobed molecule and concave on one surface, the concavity occupied by the detachable pro-part. When in contact with acid, the pro-part detaches exposing the concavity, the active site for enzyme action. The enzyme attaches to its substrate at this point and cleaves it. Acid (pH < 6) is required to convert inert pepsinogen to active pepsin but once converted, the pepsin continues the autocatalytic process sustaining the cascade in the absence of acid [3].

### 4.2. Pepsin: Isoenzymes

**Figure 1:**
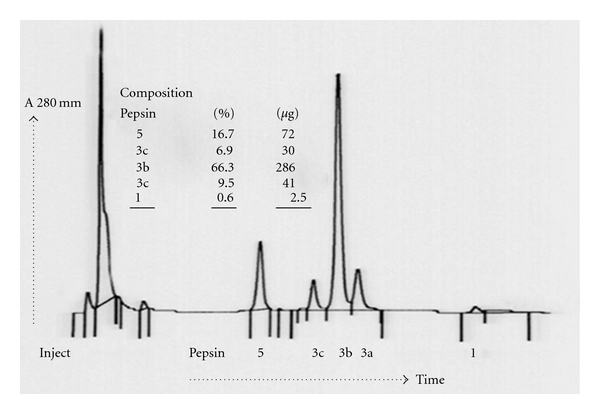
Example of pepsin profile from human gastric juice purified by HPAEC.

Pepsin has traditionally been studied by gel electrophoresis of gastric juice and tissue homogenates, which typically shows eight zones of lysis to which various names and designations have been applied based on their electrophoretic mobility [21, 26]. The pattern reflects the fact that pepsin is not a single molecule but encompasses a family of isoenzymes which structurally are similar. Today, the pepsin isoenzymes in gastric juice can be separated by high-performance anion exchange chromatography (HPAEC) using chloride counter ion gradient elution (Figure 1). Each pepsin isoenzyme has its own “optimal pH level” when its action is at a maximum, thus ensuring digestion across a wide range of gastric pH (see Table 1).

### 4.3. Pepsin: pH and Stability

The activity and stability of the enzyme is closely related to the prevailing pH of its environment, a relationship investigated over the last 40 years using different sources of pepsin, various substrates, and changing analytical methods. The results were broadly similar, and differences were attributed to the experimental conditions. Recent studies reexamining the pH-pepsin relationship in conditions of low acidity have given important new insights into enzyme stability and activity which have major clinical significance (and are discussed in detail further on).

Individual pepsin isoenzymes were noted to be stable for 24 hours even at body temperature but were ultimately degraded by autocatalysis if stored at its pH optima. In contrast, a mixture of isoenzymes, as would be found in gastric secretion, proved to be more stable [22]. For example, purified porcine pepsin was irreversibly denatured at pH 7.1, whereas peptic activity of human gastric juice persisted until exposed to pH 7.8. [27]. A “dormant” phase was observed between pH 6 and 8 when the enzyme was inactive but intact, hence it could be activated on return to pH <6 [28].

### 4.4. Pepsin: pH and Enzyme Activity: The Traditional View

It has long been known that pepsin is at its most active at pH 2 to 3, and activity is declining as acidity diminishes [29]. Emerging evidence has shown that refluxate reaching the extraesophageal areas is characterised by low acidity or none at all in those on high-dose PPI treatment. Such conditions have been widely regarded to destroy pepsin or to render it inactive, hence the scepticism that pepsin played a significant role in damaging extra-esophageal tissues. Recently, however, important new information challenges such beliefs.

### 4.5. Pepsin: pH and Enzyme Activity: The Emerging Evidence

This interrelation has recently been reassessed, now using pepsin isoenzyme 3B purified from human gastric juice [30]. It is the largest fraction of pepsin and accounts for 70% of the total enzyme effect (Table 1), for which it serves as a good marker. The assay conditions were designed to resemble those that might be expected in the human larynx in laryngopharyngeal reflux (LPR), namely, little or no acidity (pH 6.8), when pepsin would be inactive unless reactivated by subsequent acidic reflux. The enzymatic activity was measured by the rate of hydrolysis of a synthetic peptide substrate. The isoenzyme activity was at ~80% of its maximum when measured at pH 1.5 and reached its peak at pH 2. Thereafter, it declined to ~45% at pH 4.5, ~40% at pH 5, fell to ~10% at pH 6, and ceased altogether by pH 6.5. The stability of the isoenzyme was then explored having first incubated it at 37°C for 24 hours at various pH levels, ranging from 2 to 8, and assaying at pH 3.0. The enzyme stored at pH 7.0 was inactive but stable, evidenced by the observation that ~80% of its activity was recovered when reassayed at pH 3.0.

### 4.6. PPI Treatment: Effect on Pepsin Concentration

Numerous studies in man have examined the effect of PPIs on gastric acid secretion but only few on pepsin. An example is a study on the effect of high-dose omeprazole (60 mg daily for nine days) in eight healthy volunteers in whom the volume of gastric secretion and output of acid and pepsin was measured [31]. Acid secretion fell markedly from a mean of 5.4 to 0.3 mmol/h, and the volume decreased substantially from 132 to 36 mL/h. The mean pepsin output, however, fell only modestly, from 126 to 101 mg/h, but because of the reduced volume, its *concentration* rose from 90 mg to 290 mg per 100 mL.

This study, like most others on gastric secretion, relied on the measurement of stomach contents aspirated through a nasogastric tube. A novel noninvasive approach was recently used to measure gastric volume by magnetic resonance imaging [32]. Unlike the study cited earlier [31], the reduction in the volume of gastric secretion on PPI was only 12%, hence the concentration of pepsin would have increased only slightly. The clinical significance of these contrasting findings is discussed further on.

### 4.7. PPI Treatment: pH, Pepsin Activity, and H. Pylori

The PPIs commonly used today (e.g., omeprazole) on conventional dosing (single 20 mg dose in the morning) can elevate gastric pH to ≥6 but only for short periods; for much of the time, the pH is around 4 to 5 [6] and falls at night when acid secretion breaks through. Thus, for the majority of 24 hours, pepsin in gastric juice is still active or dormant but stable, hence capable of reactivation when acidity returns.

High-dose PPI treatment (e.g., omeprazole 40 mg twice daily) has a greater effect and is longer lasting, and the newer PPIs (e.g., tenatoprazole) [33] may enhance this. These conditions may keep pepsin inactive, but it seems unlikely that the pH will be elevated to levels which will result in any substantial degradation of the enzyme.

The presence of *H. pylori* increases the effect of PPI, a feature sometimes overlooked yet likely to have a bearing on the efficacy of PPI therapy in the uninfected or in those in whom the organism has been eradicated. Several studies confirm this, an example being the seminal investigation carried out in DU patients [34]. Here, the median 24-hour intragastric pH when PPIs were not used was similar before and after *H. pylori* eradication, 1.0 and 1.1, respectively. On omeprazole 20 mg, however, there was a major difference in pH, 5.5 before eradication but only 3.0 after it. The significance is that whilst the majority of DU patients are infected with *H. pylori,* its prevalence is much lower in GERD patients (and similar to that in the general population), hence theoretically, PPIs might have a lesser effect.

### 4.8. PPIs, Pepsin, and Reflux: Clinical Significance

PPI therapy suppresses acid profoundly, has a variable effect on the volume of secretion (as indicated earlier) which is difficult to explain, but does *not* reduce the frequency of reflux episodes [35]. When volume is reduced only slightly [32], much fluid remains in the stomach and is available to reflux, carrying pepsin to the extraesophageal areas. When volume is reduced substantially, the concentration of pepsin rises [31] but reflux continues [35], although less is available to reflux, what reaches the extraesophageal areas is rich in pepsin, hence is damaging.

### 4.9. Pepsin, pH, and Cell Damage

Pepsin is refluxed to the extraesophageal areas where it adheres to the epithelium [36]. If activated by acid in the refluxate, it damages the cells but even in the absence of acid the enzyme has the capacity to damage, for, though dormant, it is stable. Two mechanisms operate. The first is by its reactivation when exposed to acid in subsequent reflux episodes. The second mechanism is independent of such reflux reacidification: it is taken up within epithelial cells by endocytosis and activated from within [37]. This remarkable observation, based on laryngeal cell studies, is a recent discovery with far reaching consequences (and is discussed further on). The significance is that refluxate always contains pepsin; even if devoid of acid (as might happen on high dose PPI treatment), the enzyme will still be damaging if reflux reaches the extraesophageal areas.

### 4.10. PPIs and Pepsin: Potential Clinical Relevance

Based on older studies, pepsin is commonly assumed to become inactive at pH ≥4 and to be denatured at pH ≥5.5, hence the widespread perception that PPI treatment renders the enzyme inactive by elevating the gastric pH. This view, we suggest, now needs to be readjusted taking into consideration the new evidence which clearly shows the enzyme retains much of its activity at pH ≥4, is still intact up to nearly pH 8, can be reactivated when exposed to acid once again but can damage cells even in the absence of acid.

In clinical practice, PPIs will continue to be used in EER, frequently in high dose, for they help some who in addition to EER symptoms also have features of classical GERD as well as the few who do not [38]. From the evidence above, however, it seems unlikely that profound acid suppression with PPIs as the sole treatment strategy will give results comparable to those with typical esophageal symptoms (heartburn and regurgitation) with or without erosive esophagitis.

## 5. The Effect of Pepsin on Epithelial Cells

In laboratory studies, pepsin swiftly breaks down protein, the basis of its chemical assay. Its effect on extraesophageal tissues is in contrast subtle and perhaps sustained, depletes the cells of its defences and threatens its viability. These changes have been demonstrated in ongoing clinical and laboratory studies by Johnston and colleagues who explored the effects of human pepsin 3B (purified from gastric juice) on laryngeal epithelium using *ex vivo* systems and cell culture studies [36, 37, 39, 40].

### 5.1. Pepsin: Entry into Epithelial Cells

Based on esophageal and laryngeal biopsies from LPR patients and from control subjects, and employing a variety of analytical methods, they made three major observations: pepsin adhered to epithelial cells, was endocytosed, and caused internal cell derangements.

Pepsin was found adherent to the surface of laryngeal epithelial cells obtained from LPR patients but not to those from control subjects [36]; the absence in the latter group is to be expected, for significant reflux had already been excluded by esophageal physiology studies. The enzyme was *not* found adherent to the esophageal epithelium (in LPR patients); this is surprising bearing in mind that to reach the larynx the refluxate has first to travel along the length of the esophagus. When active, the adherent enzyme damages the intercellular junctions and depletes proteins within the cell involved in its defence (and is discussed further on).

Inactive pepsin is taken up within the cells by endocytosis through a competitive receptor-mediated mechanism and is found in vesicles located in the region of the Golgi system [37]. Such endocytosis, the second observation, is surprising, for it seems unlikely that receptors specific for pepsin exist in laryngeal tissues. Presumably, such receptors serve some other purpose but when exposed to pepsin, they “shuttle” the enzyme into the cells.

When cells were exposed to human pepsin 3B at pH 7.4, a level at which the enzyme is *inactive*, several major changes, nonetheless, occurred affecting the inner cell structure and function [39], the third major observation. The Golgi system has a pH of ~5.5, together with its associated endosomes these process large molecules such as proteins and receptors through its slightly acidic environment. The inference is that the changes observed (see below) result from reactivation of the dormant enzyme within the cell.

The cells swelled and structural damage to the mitochondria and to the Golgi system became visible on electron microscopy within an hour and increased by 12 hours. The early damage was accompanied by increased expression of seven genes involved in cell stress and toxicity including certain heat shock proteins (as a family, the production of heat shock proteins is activated when the cell is stressed and its survival threatened) and the late changes by the decreased expression of another 18 such stress genes. The investigators also used in parallel a cell toxicity assay which measures mitochondrial activity in living cells. There was a significant increase in toxicity after pepsin exposure at pH 7.4 which correlated well with the mitochondrial changes noted on electron microscopy.

The evidence strongly argues for the following chain of events: inactive pepsin is endocytosed, is activated within the cells, and causes cell damage; this induces oxidative stress and the accumulation of free oxygen radicals which, in turn, damage mitochondria and may lead to cell death. In the experimental system used, the cells were exposed only once to pepsin, thus mimicking what might happen with an isolated episode of LPR. Though damaged, the cells were still viable at 12 hours but with repeated exposure, as would be expected in chronic LPR, the damaged cells may not survive [39].

### 5.2. Pepsin: Depletion of Cell Defences

The effect of pepsin was explored using a pig laryngeal epithelial cell model. Human pepsin 3B markedly depleted cell defences only when the enzyme was made active by the presence of acid (pH 4). In contrast, acid on its own had no effect, nor did the enzyme when rendered inactive by raising pH to 7.4 or when its activity was blocked with its inhibitor, pepstatin [30].

In a series of studies, the specific cell defence changes noted were depletion of the carbonic anhydrase isoenzyme CA3 and the stress protein Sep 70, reduction of E-cadherin, and the alteration of the subtype profile of protective mucin produced [30, 36, 40, 41].

The isoenzyme CA3 is widely expressed in tissues, including the basal layers of both esophageal and laryngeal epithelium. It mediates the rapid two-way conversion of CO_2_ and water to carbonic acid, bicarbonate, and H^+^, hence plays a key role in the regulation of cell pH. When the esophageal epithelium is exposed to acid, the isoenzyme is also expressed in the more superficial cells, thus offering greater protection to the epithelium nearest the refluxed acid. In contrast, its production remains limited to the basal layers in the laryngeal epithelium.

Sep 70, like most other stress proteins, is a molecular chaperone which regulates the correct folding and unfolding of intracellular proteins during their passage through the cell. E-cadherin is crucial for maintaining adhesion between cells, and thereby mucosal integrity and its barrier function. There are several subtypes of mucin, some more prominent in specific tissues than others: collectively, they afford protection. In chronic LPR, MUC-2, -3, and -5AC are amongst the defensive mucins depleted, and *in vitro* studies confirm pepsin interferes with their production [42]. 

### 5.3. Tissue Damage in GER and EER: A Comparison

The intensity of damage of the esophageal mucosa by acid reflux (pH < 4) is proportional to the duration of contact. A degree of reflux occurs in health, particularly after meals, but peristalsis rapidly returns the refluxate to the stomach; any residue is neutralised by bicarbonate secreted in saliva and by the esophageal mucus glands.

In striking contrast, the larynx and extraesophageal structures have no mechanism for bulk removal of damaging agents, hence they must rely on intracellular defences; but as indicated earlier, such cell defences are much depleted after exposure to reflux. Hence, even slight exposure to reflux can cause disproportionate damage.

### 5.4. The Significance of Dilated Intercellular Spaces in the Squamous Epithelium

Acid injury to the esophageal squamous epithelium results in the dilatation of the intercellular spaces, which almost double in width; these changes are visible only by electron microscopy. The phenomenon is now well established in patients with both erosive and nonerosive reflux disease [43, 44]. It was recently also observed in healthy volunteers in whom the lower esophagus was infused with only weak levels of acid (pH 5.5) that the changes are no greater when strong acid (pH 2) with added pepsin (±bile acid) was infused. The changes were widespread and occurred not only at the site of infusion, but also well away from it [45].

Such dilatation has been reproduced experimentally in rabbit epithelium exposed to acid at pH 1.1 or at pH 2 but with added pepsin [44]. These tissues had reduced electrical resistance mainly due to “leakiness” of the paracellular pathways, the “leak” in proportion to the size of dextran particles which could enter the damaged tissue. The significance is that the “leak” is physical not virtual.

These studies clearly show the sensitivity of the esophageal epithelium to even low concentrations of acid. Its *possible *relevance to EER comes from the observation that dilatation of intercellular spaces has also been noted in the laryngeal epithelium in patients with LPR [36, 46], which, unlike that in the esophagus, has not attracted attention. Whilst there is little acid in refluxate reaching the extraesophageal areas, it does contain pepsin, which may potentially gain entry into the dilated spaces in the laryngeal epithelium.

The nerve endings in the esophageal epithelium (in monkeys) are located in the intercellular spaces and are superficial, appearing at a depth of only three cell layers, a major finding [43]. These sensory nerves are thought to be chemosensitive and respond to even low levels of acid, pH 5.2 to 6.9, chronic irritation leading to secondary hypersensitisation which perpetuates symptoms. The larynx, too, is richly innervated and is exquisitely sensitive. A similar train of events may, theoretically, occur in the larynx, which prolongs the symptoms even when the stimulus is much reduced.

### 5.5. Summary

In summary, esophageal mucosal damage is mediated principally by acid, and laryngeal epithelial (and *possibly* other extraesophageal epithelium) damage by pepsin (and presumably other agents). The environmental pH in the larynx and hypopharynx is ~5.5 to 6, a level at which pepsin is only slightly active or dormant. Even when inactive, pepsin damages cells after it is endocytosed and reacidified within them. The enzyme adheres to the laryngeal epithelium and when inactive is stable and can be reactivated by acidic reflux even when the episodes are infrequent. These advances in our understanding perhaps explain the apparent paradox noted in classical observations comparing EER and GER. As little as three EER episodes per week can damage the larynx, it was noted, whereas up to 50 GER episodes of acidic reflux (pH < 4) per day can be seen in the asymptomatic individual [1, 47].

## 6. The Reach and the Nature of Reflux

### 6.1. Introduction

Reflux into the esophagus is usually imagined as fluid welling up from the stomach and/or spurting in jets which sometimes reach high, a plausible interpretation of pHmetry and impedance study results, the mental image reinforced by artistic impressions in advertisements. For gastroesophageal reflux to cause disease beyond the esophagus, however, the refluxate must self-evidently reach these areas.

Reflux into the extraesophageal areas is not a new concept. Almost two decades ago, Koufman [1] speculated on its possibility as an explanation for chronic laryngeal symptoms seen in several patients but supporting evidence was to emerge only several years later as technology advanced. The presence of refluxed material in the extraesophageal areas *suggests* it may play a role in causing symptoms from the larynx and airways, but the growing evidence that pepsin can cause damage makes the case more compelling.

These insights spurred the development of new technology to detect reflux in the extraesophageal areas. In the course of these investigations, two findings emerged almost as a “byproduct” (hence seemed to attract little notice) which are likely to change the concept of EER disease. First, the refluxate loses much of its acidity as it travels upwards (presumably by neutralisation with bicarbonate in saliva and from the esophageal mucosal glands) [48, 49]. Second, refluxate can be both liquid and aerosol [49–51].

### 6.2. How Far Up the Esophagus Does Reflux Reach?

Standard pHmetry readily detects acid (defined as pH < 4) refluxed into the lower esophagus by its single sensor stationed 5 cm above the lower esophageal sphincter (LES). Dual pHmetry, in which the second sensor is stationed in the vicinity of the upper esophageal sphincter (UES), confirmed proximal reflux does occur [52]. By this time, there was also growing interest in whether refluxate containing lesser concentrations of acid was potentially damaging. The newly developed method of combined impedance pHmetry provided the means to detect liquid reflux independent of its pH and to determine how far proximally such refluxate reaches. The principle is that fluid is a better electrical conductor than air, hence impedance (resistance) falls, the higher the acid concentration, the greater the fall. The catheter has two pH electrodes, as with dual channel pHmetry (see below), and six *pairs* of impedance (resistance) sensors, each pair comprising two sensors separated by a tiny gap. Just a film of fluid is sufficient to bridge this gap and complete the circuit. In the absence of any fluid, the sensors are exposed to air and record high impedance.

The pH electrodes are stationed 5 cm and 20 cm above the LES, whilst the impedance sensors extend from above to below the pH electrodes. The resistance sensor detecting change furthermost from the LES indicates the proximal reach of reflux. These studies have clearly demonstrated refluxate frequently rises to the proximal esophagus, the significance being it is in a position to reach extraesophageal areas [53–56]. Investigations in untreated GERD patients showed 63% of reflux episodes were acidic (and 72% of symptom episodes were associated with acid reflux). In contrast, 80% of reflux episodes on PPI were weakly acid or weakly alkaline (and most symptoms were associated with refluxate of this nature) [57].

### 6.3. Acid Reflux into the Pharynx

A recent advance is the development of a nasopharyngeal probe bearing a specially constructed pH sensor at its end (available as the Dx-pH probe http://www.restech-corp.com/). The sensor is stationed in the mid-pharynx (i.e., away from the upper esophageal sphincter), where it is kept moistened with each exhaled breath. The environmental pH detected with this method is ~5.5; set against this relatively high pH, reflux containing even only little acid is readily detected [50].

### 6.4. Reflux into the Oropharynx and Airways: The Presence of Pepsin

Pepsin has recently been detected in the saliva of patients with suspected EER using a highly sensitive immunoassay which utilises two unique monoclonal antibodies against human pepsin 3 [58]. The same immunoassay has detected pepsin in the exhaled breath in patients with chronic cough thought to be due to EER. The breath sample is captured, kept cold, and the immunoassay carried out on the condensate which forms [51].

### 6.5. The Acidity of Refluxate: Its Relevance to EER Disease

Refluxate detected in impedance studies is arbitrarily divided as acid (pH < 4), weakly acid (pH 4–7), and nonacidic (pH > 7) [59]. Reflux at pH <4 is widely regarded as being damaging to the esophagus because of its high acidity, hence pH 4–7 is less injurious, and pH >7 probably without effect. The real significance of refluxate containing low or no acid, however, is that it always contains pepsin, which potentially can be carried to the extraesophageal areas, where it damages the epithelium.

As indicated earlier, esophageal damage in typical GERD is dominated by acid; in contrast, EER disease is mediated principally by pepsin. This perhaps explains why PPIs fail to rapidly improve EER disease, unlike their effect in classical GERD.

### 6.6. The Physical Form of Refluxate: Liquid and Aerosol 

The concept that reflux may also be an aerosol has only recently emerged, at this stage more plausible (albeit persuasive) than proven. It arises from detecting acid in the pharynx and pepsin in the saliva but particularly in the exhaled breath (see below). Their presence, so far away from the stomach, is more plausibly explained if they were airborne, that is, carried in an aerosol, rather than in a column of liquid rising from the stomach.

Refluxate as an aerosol has several implications. First, as indicated above, it more plausibly explains the presence of refluxate deep in the lungs [60, 61] and in the middle ear in otitis media effusions [62] (where recently bile acids too have been identified) [63]. Second, liquid refluxate probably has higher concentrations of acid and pepsin, but an aerosol is more likely to carry these damaging agents further into the extraesophageal areas. Third, their presence confirms that refluxate has reached. When in excess, and in the appropriate clinical circumstances, the findings are arguably potentially diagnostic of EER. Finally, it draws attention to a major unmet therapeutic need, namely, the development of new approaches to more effectively decrease pepsin in refluxate.

### 6.7. GER and EER and the Role of the Sphincters: Speculation

The esophageal lumen is occupied by mucosal folds and air, both swallowed and refluxed. Impaired LES function results in excessive reflux, and presumably liquid refluxate rises in the lumen along the mucosal folds. We suggest that the air in the esophagus provides the medium through which an aerosol ascends.

The LES in health allows air from the stomach to be vented whilst minimising the escape of liquids and semisolids; such separation is less efficient in GERD, where the sphincter function is impaired. The role of the UES in reflux is less well understood, but we speculate that like the LES, it too in health can distinguish liquid from gas, holding back the former and allowing the latter to be vented. We think, however, it would be difficult to distinguish between types of gas, air that contains aerosol refluxate from air that does not. Hence, both are vented and refluxate reaches the extraesophageal areas.

## 7. Detecting Extraesophageal Reflux

### 7.1. Diagnosis: A Note

Whilst it is beyond the scope of this paper to consider the issues surrounding the diagnosis of EER in any detail, we would like to highlight the following.

### 7.2. Clinical Manifestations of EER

Today, EER is increasingly considered as a potential cause of symptoms in adults with chronic problems arising from the larynx, throat, and airways. Changes are often seen in the larynx, but there is no feature characteristic of damage by EER. Furthermore, EER when present may be one of several contributory factors, for example, smoking.

### 7.3. Technology Currently Available

Impedance pHmetry clearly identifies liquid reflux and indicates how far proximally it reaches. Here, it is assumed that liquid is poised to penetrate into the extraesophageal areas hence EER can be *inferred*. Conventionally, one looks to correlation between proximal reflux episodes and symptoms as evidence of a causative link; but bearing in mind that even infrequent episodes of EER can be damaging, the absence of such correlation does not necessarily exclude reflux-related damage.

Impedance pHmetry (MIIpH) has given us remarkable insights into the pathophysiology of gastroesophageal reflux; it readily detects liquid refluxate irrespective of its pH (hence, it is particularly useful when investigating patients already on PPI) and can determine how often and how far up the esophagus the refluxate reaches—but not beyond it (as yet), nor can it, as currently configured, identify aerosol reflux. To detect EER, therefore, we suggest its role is more supportive than diagnostic.

### 7.4. Emerging Technologies: Acid and Pepsin in the Pharynx, Saliva and Breath

Aerosol acid reflux into the pharynx can today be detected by the Dx-pH nasopharyngeal probe (ResTech). Diagnostic criteria have been developed, and a pH level <5.5 is regarded as abnormal [50].

Pepsin immunoassay now makes it possible to detect minute amounts of the enzyme in saliva and in exhaled breath. It has recently been adapted as a lateral flow-based test (Peptest^TM^, RD BioMed Ltd., UK), easy to use, with a detection lower limit of 16 ng/mL, and with results available within minutes. The sensitivity is only slightly lower than with conventional ELISA, which is laborious and more timen consuming. The test is noninvasive and will prove more acceptable to patients, particularly if serial assays are clinically required. Determining its clinical usefulness in diagnosis and in monitoring treatment will, however, require more extensive studies.

Venting air is normal and with it a tiny amount of aerosolised gastric content is likely to escape, hence the asymptomatic *may *have pepsin detectable in the saliva. How should such a finding be interpreted? Lessons learnt two decades ago of the diagnosis of GER by pHmetry give guidance. Some reflux of acid into the esophagus is physiological and not associated with any symptoms; that is, it is the norm. Symptoms (with or without erosive esophageal damage) develop only when reflux is excessive. Thus, the difference between health and disease is a quantitative one, that is, the *degree* of acid reflux. The same model may apply to detecting pepsin in the saliva. The test is quantitative; we *may,* therefore, find that as with GER it is the quantity of pepsin in extraesophageal areas that correlates best with disease (as opposed to the presence of a tiny amount).

## 8. Medical Therapy

### 8.1. The Place of PPIs

PPIs, dramatically effective in typical reflux, are rather less so in EER. Several clinical trials and meta-analyses have failed to show clear benefit in LPR [64] other than one single study that observed a benefit of twice daily PPI for LPR symptoms and signs [65]. Patients with asthma when considered as a group showed no discernible benefit with PPIs; the subset with GER symptoms, however, were helped [66] but, interestingly, not those with pHmetry-proven reflux alone (in the absence of GER symptoms) [67]. Patients with chronic cough in whom EER was suspected also did not benefit [68, 69].

Tenatoprazole, a new PPI with a much longer half-life than those currently available, correspondingly suppresses acid for a longer period in the 24 hours [33]. Other PPIs and potassium-competitive acid blockers are in development [5, 6]. These may prove helpful in patients with typical reflux when currently available PPIs give insufficient response; but for the reasons stated earlier, they are unlikely to make much difference in EER.

Nevertheless, the occasional patient does show improvement, generally partial, with PPIs. In the absence of really effective treatment, such anecdotes encourage continued widespread use of profound acid inhibition.

### 8.2. Antipepsins

Drugs with antipepsin activity have been used in several clinical studies in patients with peptic ulcer but were found not to be effective. Examples are amylopectin sulphate [70–72] and pepstatin [73].

### 8.3. Alginates: “Sieving Pepsin” from Gastric Secretion

Alginates, widely available for almost 40 years, have recently been shown to have a powerful effect on pepsin and bile in refluxate *in vitro *[74] and potentially offer an effective treatment [75].

Recent *in vitro* studies confirm that Gaviscon Advance (GA), a specific alginate formulation, removes ~90% of pepsin and bile in the first “reflux episode”, declining to about 50% by the tenth. Their rate of depletion was similar, suggesting a common mechanism, most probably selective binding [74].

### 8.4. Alginates: Effectiveness in EER?

Only a few studies have as yet been done, mainly in LPR, and give encouraging results.

#### 8.4.1. LPR

A UK study assessed the benefit of adding GA to standard vocal hygiene advice (control group) [75] and a USA investigation on the outcome of adding GA to preexisting PPI therapy [76]. In both, a dose of 10 mL × 4 daily was used; this is the recommended dose when the drug is used for dyspepsia and is generally used only for short periods (4 to 8 weeks) but for the trials treatment was given for six months.

Allocation to treatment in the UK study was randomised and blinded. The outcome was assessed by those unaware of the patients' treatment group. Two scoring instruments were used, both validated and semiquantitative. The reflux symptom index (RSI) measures the symptom burden and the reflux finding score (RFS) the degree of change observed on endoscopic examination.

The baseline scores were similar in both groups. RSI improved significantly in the group compared to baseline both at 2 and 6 months, but the improvement was much greater for those on GA. Treatment with GA gave a significant improvement in RSI compared to the control group at 2 and 6 months. The RFS did not change in the control group but improved significantly in the GA-treated group but only at six months; this suggests that endoscopic improvement lags behind symptom relief [75].

The USA study [76] used both RSI and RSF and, in addition, a voice-related quality of life index (QLI). Both treatment groups had similar RSI symptom scores at the start (comparable to that in the UK study) and similar degree of improvement at two months. Thereafter, however, there was no further symptom improvement in those on PPI alone, whereas the group on PPI + GA continued to make gains, the differences being significant at 4 and 6 months. This change in symptom intensity was also reflected in the RFS and QLI.

#### 8.4.2. Chronic Cough

EER is often suspected in patients with chronic cough. It is, therefore, surprising that no major study has explored the value of using GA in this group.

### 8.5. Alginates: Reengineering the Polymer?

Alginates are hydrocolloids of vegetable origin and are a structural component of marine brown algae to which it gives strength and flexibility. These hydrocolloids are polymers and have the property of forming gels, films, thickeners, and stabilisers. The polymer is composed of two monomers, mannuronate and guluronate, and differences in the properties of alginates are determined by their ratio. Added calcium binds to specific sites and stiffens the overall structure.

The remarkable properties of these natural polymers can, we suggest, be enhanced by modern polymer chemistry, making it possible to develop derivatives with more powerful and specific actions.

### 8.6. Focusing on Pepsin at Source and at Target

The treatment of EER today is as we were circa 1970 for the treatment of peptic ulcer: the need to reduce acid secretion powerfully was increasingly recognized, but the only drugs then available were antacids (the use of anticholinergics, which also reduced acid secretion, restricted by their side effects). Today, we increasingly recognise the importance of pepsin; alginates help—but effective treatment will probably require substantial if not profound suppression of pepsin secretion (as was achieved for acid with the H_2_RAs initially and then with PPIs).

Pepsinogen is secreted by the gastric peptic cells. A great deal is known about the intricate physiology of acid secretion from the parietal cells but not nearly as much on the regulation of pepsinogen secretion. To powerfully reduce the secretion of this proenzyme at the cellular level requires deeper understanding of mechanisms and probably the development of specific inhibitors: this, we suggest, is an avenue for the future.

Minimising or halting the damage caused when the enzyme is adherent to the extraesophageal epithelium and positioned to be endocytosed requires a different strategy [37, 39]. Hence, the development of an irreversible pepsin inhibitor has recently been mooted.

We speculate that these two strategies, if and when developed, are likely to be used together in troublesome EER disease, the irreversible inhibitor against pepsin already adherent, and a secretion inhibitor to markedly reduce pepsinogen secretion and hence pepsin at source, thereby preventing further damage.

### 8.7. Surgery

Antireflux surgery for GERD gives very good results in the majority of patients who are carefully selected for this operation, and extensive clinical and trial experience has defined its role in patient management. Because gastroesophageal reflux is at the root of both GERD and EER, it is tempting to presume antireflux operation will also give similar benefit in extraesophageal reflux. There are indeed anecdotal instances where operation has helped individual patients but as yet this cannot be generalized, for there are substantial differences between the two conditions.

There are as yet no specific selection criteria with which to identify those with EER who are likely to benefit from surgery. The development of such criteria for GERD and the optimisation of antireflux surgery developed over the last two decades of the 20th century. It is likely to take several years for a similar position to be reached for the surgical treatment of EER.

## 9. Conclusion

Knowledge of GERD emerged in the last third of the 20th century, as growth accelerating after the development of PPIs, which triggered many studies on the pathophysiology and dominated treatment. Knowledge of EER, a part of the reflux spectrum but with distinct characteristics, is still emerging and, like GERD, may prove to be a worldwide problem.

The presence of excessive acid in the esophagus is crucial for the development of GERD symptoms and mucosal damage, hence the benefit of PPIs which selectively and profoundly inhibit it. When extended to EER, however, the results are poor. Emerging knowledge now provides a persuasive explanation: EER is much more dependent on pepsin-mediated damage in the laryngeal and airway mucosa than with acid.

It was the recognition that acid might play a major role in peptic ulcer which led to the development of the H_2_RA and subsequently to PPIs, the real power of which was to be found in GERD, where profound acid reduction is important. We believe the recognition now of the crucial role of pepsin in EER may, in turn, stimulate the development of drugs which specifically target this molecule. This may radically enhance our knowledge and management of this condition.

## Figures and Tables

**Table 1:** The active pepsin isoenzymes in man.

Pepsin isoenzyme	Proportion of total pepsin	Substrate: pH optimum range	Molecular weight (Da)	Comments
1	<5%	Haemoglobin: 1.9 Collagen: 3.0 Mucin: 2.0	43810	The proportion of pepsin 1 rises to 23% in DU Mucolytic pepsin Complexed with carbohydrate

2	<1%	Haemoglobin: 2.1	39950	

3	80%	Haemoglobin: 2.4–3.1	37150	Pepsin 3 is a complex of isoenzymes. The assay measures their combined effects.
3A 3B 3C	6% 70% 4-5%	Haemoglobin: 3.2		3A is structurally similar to 3B but is phosphorylated

5 (Gastricsin)	6-7%	Haemoglobin: 2.0–3.6 (maximum at 3.2) Mucin: 3.5–5	31620	Stable up to pH 7.3

“Pepsin 4” is a complex of pepsin and an inhibiting peptide, hence, it does not appear in the list of active pepsin isoenzymes.

“Pepsin 6” is the remnant of a zymogen, in all probability pepsinogen, hence it too does not feature in the isoenzyme list.

“Zone 7” was found to be a cathepsin.

References [21–25].

## Abbreviations

DU: Duodenal ulcer
EER: Extraesophageal reflux
GA: Gaviscon Advance
GERD: Gastroesophageal reflux disease
H_2_RA: Histamine H_2_ receptor antagonists
HCl: Hydrochloric acid
*H. pylori:*: *Helicobacter pylori*
LES: Lower esophageal sphincter
LPR: Laryngopharyngeal reflux
PPI: Proton pump inhibitor
QLI: Quality of life index
RFS: Reflux finding score
RSI: Reflux symptom index
UES: Upper esophageal sphincter.
